# A Peel Test Method to Characterize the Decay Law of Prepreg Tape Tack at Different Temperatures

**DOI:** 10.3390/ma17102449

**Published:** 2024-05-19

**Authors:** Jiaqi Shi, Wang Wang, Yuequan Wang, Junwei Qi, Jun Xiao

**Affiliations:** College of Material Science and Technology, Nanjing University of Aeronautics and Astronautics, Nanjing 210016, China

**Keywords:** handling life, tack, prepreg, composite

## Abstract

The tack of prepreg is a key factor affecting the automatic tape laying process. During the manufacturing process of large composite parts, prepreg material may be stored at room temperature for several days, resulting in a decrease in its tack. In this study, a new tack test tool was designed, and the decay rate of prepreg tack at different temperatures was tested. We proposed a prepreg tack decay model, which assumes that the main factor in tack decay is the reduction in resin chain activity during storage. The maximum deviation between the model calculation results and the experimental results of the tack decay rate is 9.7%. This study also proposed a new statistical unit for prepreg tack, which can establish the relationship between the tack of prepreg and its remaining storage time and reduce prepreg management costs.

## 1. Introduction

Composite materials can enhance the fuel efficiency of commercial aircrafts; thus, their mass fraction becomes a benchmark for assessing the manufacturing level of commercial aircraft. Currently, the main automated production processes for producing aircraft composite parts are automated fiber placement (AFP) and automatic tape laying (ATL). The ATL process is often used in the production of large wings. Currently, the ATL process for wings employs thermosetting prepreg materials, and the tack of thermosetting prepregs is a critical factor affecting the manufacturing process [1]. Prepregs are stored at room temperature during production, and large parts may cause this storage to approach the handling life limit of the prepreg [2]. Therefore, it is necessary to study the decay of prepreg tack with storage time. However, the tack of prepreg as a material property lacks clear quantification testing standards and mechanistic models [3].

Initial studies on the tack of prepregs used pressure-sensitive adhesive testing methods, such as ASTM D6195 adhesive loop tack strength testing [4] and ASTM D3121 rolling ball test [5]. However, both of these test methods are difficult to accurately measure prepreg tack. Dubois et al. [6] used a probe method to measure the tack of prepreg and analyzed the load curves during bonding and separation. However, this method is essentially a modified version of measuring tack using fingers [7]. Ahn et al. [8] measured the bonding strength between prepregs by compressing and separating multiple layers of prepreg. This test method of removing the prepreg as a whole is very different from the process of peeling the prepreg from the laminate. Nguyen et al. [9] designed a single-lap-based adhesion measurement method that is suitable for evaluating the connection strength of lap joints used for prepreg extension in automated production. Böckl et al. [10] developed a friction-based tack test method that measures the lateral friction of the prepreg tape passing through a measuring roller. The test results can be used to monitor the automatic placement process, but the transferability of the test results to the prepreg bonding strength remains to be verified. Crossley [11] designed a tack-measuring fixture that measures the force required to peel a prepreg from a stainless steel plate. Endruweit et al. [12,13] used the same method to study the effect of tape laying process parameters on prepreg tack. Brooks [14] also used the peeling method to measure the tack of prepreg. His test method had an independent sample preparation device, and he tested the peeling force using a universal testing machine. The peel test method designed by Crossley and Brooks is closer to the reverse process of tape laying and is more suitable as a quantitative test standard for prepreg tack.

Researchers found that the tack of prepregs decreased with aging until it could not be measured [15,16]. The aging process is also accompanied by changes in the properties of the resin. Researchers have found that high-pressure liquid chromatography shows that the resin composition will change during the aging process [17]. Moreover, aging will increase the glass transition temperature (Tg) of the prepreg [18], and Fourier-transform infrared spectroscopy results also prove that the functional groups of the resin will change during the aging process [19]. The researchers not only tested the tack decay of prepregs but also proposed different models for the mechanism of tack decay. The tack decay of prepreg is a change in its surface properties [20]. Unlike adhesives that form chemical bonds with the bonded surfaces [21,22], the prepreg tape laying process only takes a few seconds, which is not enough time for the reaction to form a chemical connection. The adhesive force comes from the van der Waals force and hydrogen bonds generated by the resin chains [23,24]. The changes in the resin during the aging process will lead to a decrease in the van der Waals force and hydrogen bond strength between the prepreg resin molecules, thereby reducing the tack.

This study designed a tack testing device based on the automatic tape laying machine, and measured the tack changes of prepreg during the aging process. Based on the test results, a prepreg tack decay model based on resin molecule movement and the Arrhenius equation was proposed. Finally, this study proposes a new tack measurement unit to reduce the prepreg handling life management cost.

## 2. Experimental Section

### 2.1. Experimental Device

**Figure 1:**
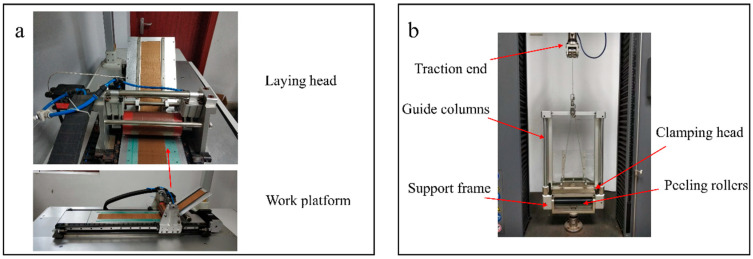
Test device. (**a**) Automatic tape-laying platform; (**b**) peeling test fixture.

The test device of Crossley is a peeling fixture that needs to be operated by a universal testing machine. Therefore, the speed, pressure, and other parameters will be limited by the performance of the testing machine. Brooks used a specialized sample preparation device to overcome the parameter limitation, but there was no fixture constraint during the peeling process in his test method, so the measured load curve fluctuated greatly. This study combines the advantages of the two methods, specialized sampling device that allows the parameters to be set closer to the ATL process, and the design of a peeling fixture to make the load curves less noisy. The test device is shown in Figure 1, which includes an automatic tape laying device and a peeling fixture. The automatic tape laying device was designed based on the basic functional requirements of the ATL machine and consists of a work platform and a laying head. The work platform surface can fix the prepreg by negative pressure, and a weighing sensor is placed at the bottom of the work platform to provide feedback on the laying pressure. The laying head consists of a 60 mm diameter rubber pressure roller, cylinder, and support frame. The hardness value of the pressure roller is 30A–35A. The cylinder provides uniform and controllable pressure to the prepreg tape being laid through the pressure roller. The laying head is installed on the guide rail of the work platform and is driven by a motor, allowing the platform to lay prepreg up to a maximum width of 150 mm. The entire system is controlled by a PLC (Programmable Logic Controller). During the sample preparation process, the prepreg to be bonded is placed on the platform and in the guide groove, and the laying pressure and speed are set. The device then starts to complete the bonding. In this study, the laying speed was set to 50 mm/s, and the laying pressure was set to 500 N.

To solve the problem of high noise from the force sensor caused by sample oscillation during peeling, this work designed a special peeling fixture, as shown in Figure 1b. The fixture consists of a support frame, clamping head, traction end, guide columns, and peeling rollers. The clamping head is connected to the traction end via a wire rope pulley system. After clamping the prepreg, the universal testing machine pulls the traction end to peel it. During the peeling process, the guide column on the frame can constrain the prepreg to only move vertically, the peeling roller constrains the peeling angle of the prepreg, and the transparent limiting plate constrains the swinging direction of the prepreg.

The aging experiment was conducted using the CH225R-type environmental test chamber from Tuode Environmental Testing Equipment Co., Ltd, Dongguan, China. This chamber has a temperature control accuracy of ±1 °C within the range of −10 °C to 100 °C, and a humidity control accuracy of ±5% RH.

### 2.2. Experimental Materials

This paper tests three types of carbon fiber epoxy uni-directional prepregs that are widely used in the aerospace industry, namely T, C, and H. The T-type prepreg has a resin content of 35% by weight, while the C-type and H-type prepregs have a resin content of 34% by weight. The manufacturer of T-type prepregs has provided a handling life table, which shows the decay rate of the handling life at different aging temperatures, as presented in Table 1.

### 2.3. Experiment Procedure

To evaluate the tack decay rate of T-type prepreg at different aging temperatures, an experimental matrix was established, as presented in Table 2. Since the humidity in the production workshop was constant and the manufacturer’s data did not include changes in humidity, it was not considered a variable in this study. To ensure significant differences among the test values, the most severe value in Table 1 was chosen as the experimental condition, and the humidity was set at 65% RH. To guarantee uniform changes in the life value between adjacent test points, aging times at various temperatures were calculated based on the life decay rate ratio.

**Figure 2:**
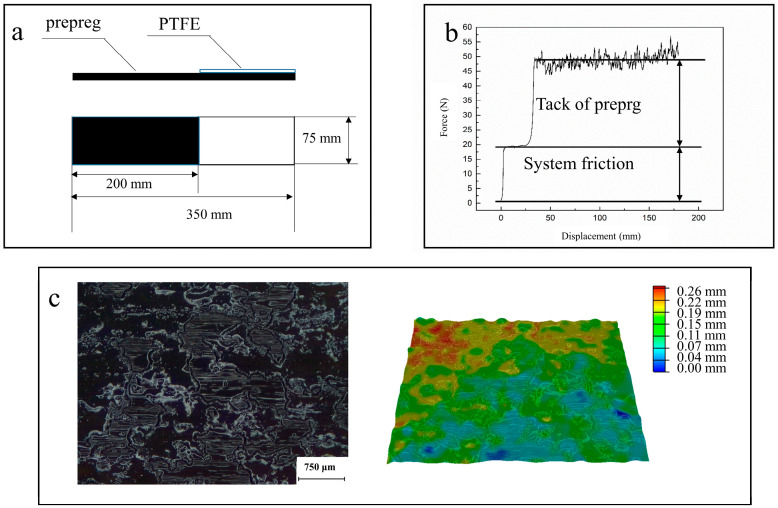
(**a**) Dimensions of the specimen. (**b**) Load–displacement curve for T stripping. (**c**) Inhomogeneous distribution of the resin.

The prepregs were cut to the size shown in Figure 2a and then placed into the CH225R-type environmental test chamber. After aging, the prepregs were transferred to a constant temperature room at 23 °C for testing following sample preparation on the automatic lay-up platform. The samples were clamped on the designed peeling fixture and tested. The load–displacement curve obtained from the experiment includes a low plateau region and a high plateau region, as shown in Figure 2b. The former is due to the frictional force generated by the entire system, and the latter is the result of the former plus the tack of the prepreg. Figure 2c shows that the resin distribution is inhomogeneous, and therefore the loads do not present a straight line. Contamination of the bonding surface was prevented during the aging process and sample preparation. To minimize sample aging during the waiting period, the nine samples were divided into three groups for testing, and each point on the graph was obtained from the average of nine experiments.

## 3. Results Analysis and Discussion

### 3.1. Analysis of Aging Results

**Figure 3:**
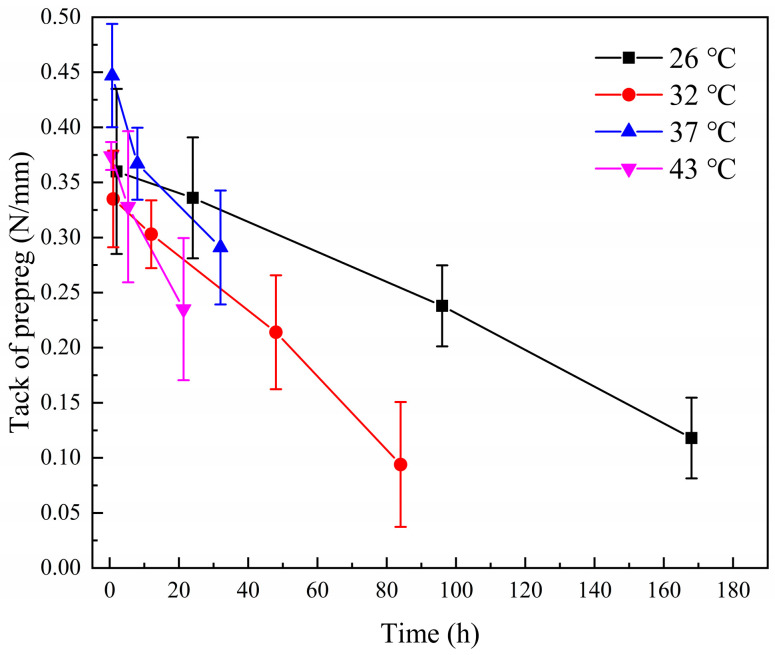
Decay of tacks of T-type prepreg at different aging temperatures.

The tack decay curve of the T-type prepreg after aging is shown in Figure 3. Due to the long experimental time, it is impossible to use the same batch of prepreg to complete all aging test points within its process life. Therefore, the experiment only ensures that the same batch of prepreg is used at the test points of each curve to ensure the continuity of the aging process. The slope of the tack decay rate after aging at different temperatures was fitted, and it was found that the decay rate increased with increasing temperature. We calculated the ratio of decay rates at different temperatures relative to 26 °C. The ratio of decay speed at different temperatures was 1.0 (26 °C):1.9 (32 °C):3.1 (37 °C):4.9 (43 °C). The Nash–Sutcliffe model efficiency coefficient test formula for the fitting results is as follows:

(1) $$R^{2}=1-\frac{SS_{res}}{SS_{tot}}$$

(2) $$SS_{res}=\sum_{i}{\left(y_{i}-{\hat{y}}_{i}\right)}^{2}$$

(3) $$SS_{tot}={\sum_{i}\left(y_{i}-\bar{y}\right)}^{2}$$

where *y* is the true value, *ŷ* is the predicted value, $\bar{y}$ is the mean value, *R*^2^ is the coefficient of determination, *SS_res_* is the sum squared regression, and *SS_tot_* is the total sum of squares.

The results of *R*^2^ are shown in Table 3, which shows a good fit for the decay results. The deviations between the ratio of the fitted results and the manufacturer’s results were −5% (32 °C), 3.3% (37 °C), and 8.9% (43 °C), respectively. While it is not possible to know the manner in which the manufacturer evaluates the prepreg decay rate, the methodology used herein yields similar results.

### 3.2. Tack Decay Model

In this study, a tack decay model of prepreg based on resin chain diffusion and the Arrhenius equation was proposed. Wool [25] proposed a resin movement theory for thermoplastic resin welding. The model assumes that the depth of resin chain diffusion determines the joint strength, and the movement of the resin chain can be equivalent to a random walk chain diffusing in a tube. The resin chain movement ability is affected by the molecular mass of the resin. The prepreg bonding process also involves the formation of van der Waals forces and hydrogen bonds between resin chains after resin diffusion. Therefore, the model proposed by Wool is also applicable to uncured thermosetting resins. The resin chain movement model is as follows:

*σ_d_* = *qn*_0_*χ* (4)

where *σ_d_* indicates the force between resin chains, *q* is a constant, *n*_0_ means the total number of constraints per unit volume of the virgin bulk material, and *χ* means chains self-diffusing across the interface to an interpenetration depth.

**Figure 4:**
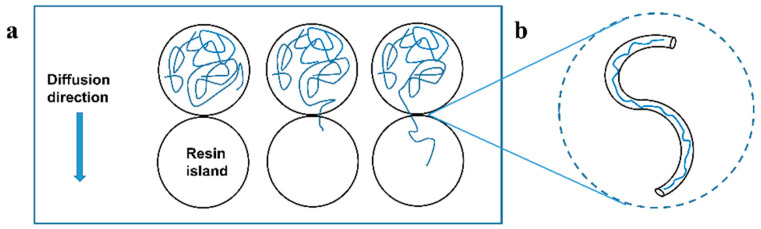
(**a**) Processes of resin movement. (**b**) The model of a random walk chain diffusing in a tube.

The diffusion motion of the molecular chain can be equivalent to the model of a random walk chain diffusing in a tube [26,27], as shown in Figure 4.

The relationship of random penetration depth can be obtained as follows:

(5) $${\langle \chi^{2}\rangle }^{1/2}=\alpha {\left(2D_{c}t\right)}^{1/4}$$

where α is the coefficient, $D_{c}$ is the diffusion coefficient, and t is the diffusion time.

Since $D_{c}$ depends on the molar mass M of the resin,

(6) $$D_{c}∼1/M$$

The diffusion capacity of the resin chain depends on its molecular mass, and cross-linking reactions of the resin will increase its molecular mass and thus reduce its diffusion capacity. Therefore, the rate of decay of prepreg tack at different temperatures should be positively correlated with the rate of reaction of the resin. The reaction rate of the resin at different temperatures follows the Arrhenius equation.

(7) $$k=Ae^{-E_{a}/RT}$$

where k is the reaction rate constant, *A* is the Arrhenius constant, *E_a_* is the activation energy of the reaction, *R* is the gas constant, and *T* is the thermodynamic temperature.

**Figure 5:**
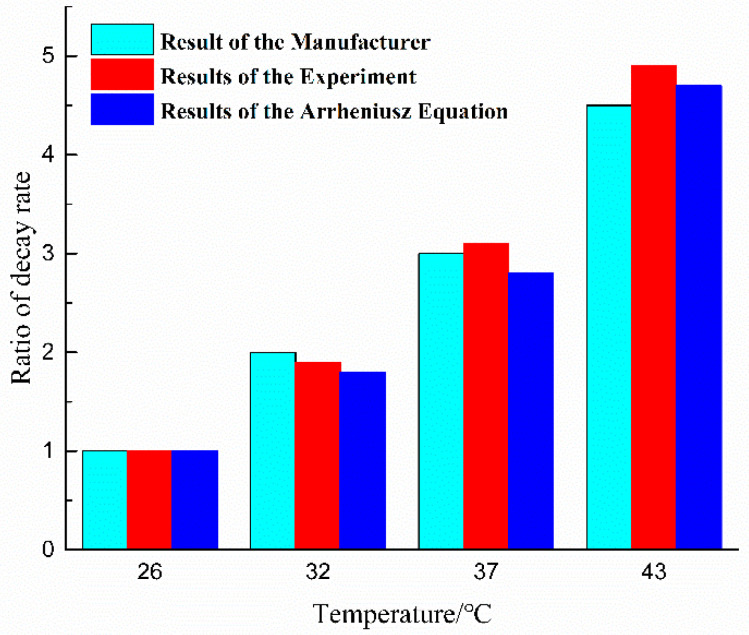
Ratios of tack decay rates obtained by different methods.

Janković [28] used the invariant kinetic parameters method to obtain a reaction activation energy of 71.4 kJ/mol for the T-type prepreg’s resin. The ratios of the reaction rates calculated using the Arrhenius Equations (7) were 1.0 (26 °C), 1.8 (32 °C), 2.8 (37 °C), and 4.7 (43 °C), respectively. This result deviated from the data provided by the manufacturer of the T-prepreg by −10.0% (32 °C), −6.7% (37 °C), and 4.4% (43 °C), and from the experimental results by −5.3% (32 °C), −9.7% (37 °C), and −4.1% (43 °C). This result indicates that the results of tack decay are consistent with the rate of chemical reaction and indirectly demonstrates that the curing reaction of the resin is a factor in the tack decay of the prepreg. The ratios of decay rates are shown in Figure 5.

### 3.3. The Unit of Handling Life

**Figure 6:**
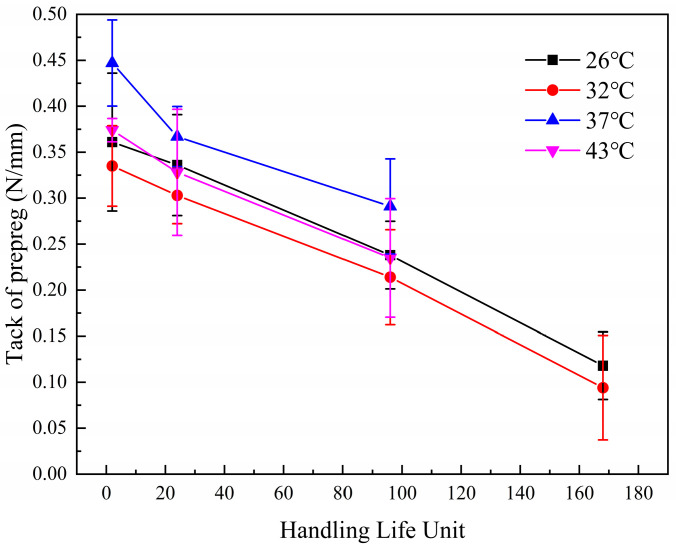
The tack of T-prepregs at different aging temperatures decays with handling life unit.

In order to establish a relationship between the change in tack value of the prepregs and time, the magnitude of the decrease in tack for one hour of aging at 26 °C/65% RH was recorded as a unit of handling life. A 26 °C/65% RH meets the environmental conditions of most composite material workshops. Using the unit of handling life as the horizontal coordinate, the curve of tack change with the unit of handling life is plotted. Figure 6 shows the change in tack of T-type prepregs with handling life unit. The four curves in the figure show the same slope, which indicates that when the handling life unit is the horizontal coordinate, the tack decay at different temperatures shows the same law. The unit of handling life allows the composite manufacturer to translate the available tack range of the prepreg into the maximum operating downtime at 26 °C/65% RH. Even if the prepreg is subjected to complex temperature aging during downtime or transport, the remaining handling life can be determined by measuring the difference between the tack of the prepreg and the minimum tack permitted for production. The handling life unit establishes a relationship between tack and remaining shelf life and reduces the cost of managing prepregs for manufacturers.

### 3.4. Model Adaptability

**Figure 7:**
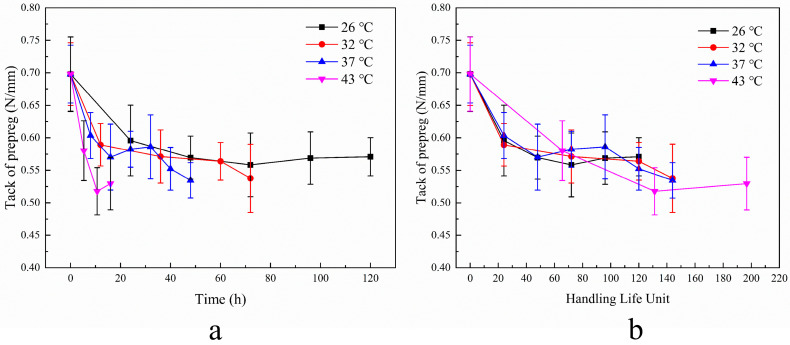
Decay of tack of C-type prepreg at different aging temperatures. (**a**) Change in tack over time. (**b**) Change in tack with handling life unit.

**Figure 8:**
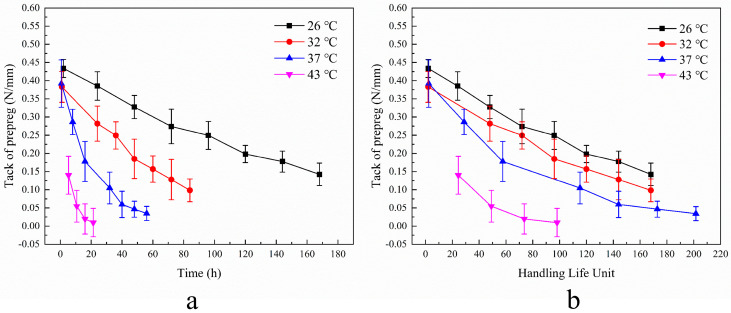
Decay of tack of H-type prepreg at different aging temperatures. (**a**) Change in tack over time. (**b**) Change in tack with handling life unit.

To evaluate the method’s applicability to other products, C-type and H-type epoxy prepregs were also tested using the method, and the results are presented in Figure 7a and Figure 8a. The decay rates of C-type prepreg at different aging temperatures are 1 (26 °C):2 (32 °C):3 (37 °C):12.3 (43 °C), while those of H-type are 1 (26 °C):2 (32 °C):3.6 (37 °C):4.6 (43 °C). The decay curves for each prepreg are parallel when using the handling life unit as the horizontal coordinate, as shown in Figure 7b and Figure 8b.

The test method in this paper can also obtain the results of its tack decay rate at different temperatures on H-type and C-type prepregs. It proves that this method has good adaptability between different types of prepregs. The *R*^2^-test results of the above two prepregs are shown in Table 4. It can be seen from the *R*^2^ results that the linear fitting effect of C-type prepreg is worse than that of H-type prepreg. The reason is that with the increase in aging time, the attenuation amplitude of C-type prepreg gradually decreases. It has been found that C-type prepregs add thermoplastic components to the resin in order to improve toughness [29], and therefore may have an effect on the change in tack, which requires further study.

## 4. Conclusions

In this paper, a new prepreg tack test device is designed. The device was used to test the law of prepreg tack decay over time at different temperatures. Based on the experimental results, a prepreg tack decay model was proposed. The model assumes that the resin reaction reduces the mobility of the resin chain, resulting in a decrease in tack. The reaction rates at different temperatures were calculated using the Arrhenius equation, and the maximum deviation from the decay rate measured by the tack experiment was 9.7%. We proposed the concept of a handling life unit, which is used as a life unit by measuring the value of tack lost by aging a prepreg for one hour at the operational ambient temperature. This concept allows for the establishment of a relationship between prepreg tack and remaining shelf life, reducing the administrative costs for manufacturers.

## Figures and Tables

**Table 1:** Manufacturer’s recommended handling life table for T-type prepregs.

Temperature	Handling Life Decay Rate Ratio
<26 °C	1
26~32 °C	2
32~37 °C	3
37~43 °C	4.5

**Table 2:** Aging test table.

Aging temperature/°C	43	37	32	26
Aging time/Hour	5.3	8	12	24
	21.3	32	48	96
	37.3	56	84	168
	53.3	80	120	240
	69.3	104	156	312

**Table 3:** *R*^2^ test for T-type prepreg.

Temperature	T-Type Prepreg of *R*^2^
26 °C	0.996
32 °C	0.995
37 °C	0.905
43 °C	0.989

**Table 4:** *R*^2^ test for H-type prepreg and C-type prepreg.

Temperature	H-Type Prepreg of *R*^2^	C-Type Prepreg of *R*^2^
26 °C	0.981	0.547
32 °C	0.983	0.705
37 °C	0.900	0.707
43 °C	0.862	0.793

## Data Availability

Dataset available on request from the authors.

## References

[1] Putnam J.W., Seferis J.C., Pelton T., Wilhelm M. (1995). Perceptions of Prepreg Tack for Manufacturability in Relation to Experimental Measures. Sci. Eng. Compos. Mater..

[2] Miller S.G., Sutter J.K., Scheiman D.A., Maryanski M., Schlea M. Study of Out-Time on the Processing and Properties of IM7/977-3 Composites. Proceedings of the SAMPE.

[3] Budelmann D., Schmidt C., Meiners D. (2020). Prepreg Tack: A Review of Mechanisms, Measurement, and Manufacturing Implication. Polym. Compos..

[4] Pierik R. Experimental Setup and Method for the Characterization of Ply-Ply Adhesion for Fiber-Reinforced Thermoplastics in Melt. Proceedings of the 26th International ESAFORM Conference on Material Forming, ESAFORM 2023.

[5] Jois K.C., Mölling T., Schuster J., Grigat N., Gries T. (2024). Towpreg Manufacturing and Characterization for Filament Winding Application. Polym. Compos..

[6] Dubois O., Le Cam J.B., Béakou A. (2010). Experimental Analysis of Prepreg Tack. Exp. Mech..

[7] Mohammed I.K., Charalambides M.N., Kinloch A. (2015). Modelling the Interfacial Peeling of Pressure-Sensitive Adhesives. J. Nonnewton. Fluid Mech..

[8] Ahn K.J., Seferis J.C., Pelton T., Wilhelm M. (1992). Analysis and Characterization of Prepreg Tack. Polym. Compos..

[9] Nguyen C.D., Krombholz C. Influence of Process Parameters and Material Aging on the Adhesion of Prepreg in AFP Processes. Proceedings of the ECCM 2016—Proceeding of the 17th European Conference on Composite Materials.

[10] Böckl B., Jetten C., Heller K., Ebel C., Drechsler K. Online Monitoring System for the Tack of Prepreg Slit Tapes Used in Automated Fiber Placement. Proceedings of the ECCM18—18th European Conference on Composite Materials.

[11] Crossley R.J., Schubel P.J., Warrior N.A. (2011). Experimental Determination and Control of Prepreg Tack for Automated Manufacture. Plast. Rubber Compos..

[12] Endruweit A., Choong G.Y.H., Ghose S., Johnson B.A., Younkin D.R., Warrior N.A., De Focatiis D.S.A. (2018). Characterisation of Tack for Uni-Directional Prepreg Tape Employing a Continuous Application-and-Peel Test Method. Compos. Part A Appl. Sci. Manuf..

[13] Endruweit A., Ghose S., Johnson B.A., Kelly S., De Focatiis D.S.A., Warrior N.A. Tack testing to aid optimisation of process parameters for automated material placement in an industrial environment. Proceedings of the 21st International Conference on Composite Materials.

[14] Brooks J.R., Platt P.R. (1996). 5513537 Method and Apparatus to Determine Composite Prepreg Tack. Compos. Part A Appl. Sci. Manuf..

[15] Cole K.C., Noël D., Hechler J.-J., Cielo P., Krapez J.-C., Chouliotis A., Overbury K.C. (1991). Room-temperature Aging of Narmco 5208 Carbon-epoxy Prepreg. Part II: Physical, Mechanical, and Nondestructive Characterization. Polym. Compos..

[16] Blass D., Kreling S., Dilger K. (2017). The Impact of Prepreg Aging on Its Processability and the Postcure Mechanical Properties of Epoxy-Based Carbon-Fiber Reinforced Plastics. Proc. Inst. Mech. Eng. Part L J. Mater. Des. Appl..

[17] Scola D.A., Vontell J., Felsen M. (1987). Effects of Ambient Aging of 5245C/Graphite Prepreg on Composition and Mechanical Properties of Fabricated Composites. Polym. Compos..

[18] Hübner F., Meuchelböck J., Wolff-Fabris F., Mühlbach M., Altstädt V., Ruckdäschel H. (2021). Fast Curing Unidirectional Carbon Epoxy Prepregs Based on a Semi-Latent Hardener: The Influence of Ambient Aging on the Prepregs Tg0, Processing Behavior and Thus Derived Interlaminar Performance of the Composite. Compos. Sci. Technol..

[19] Jones R.W., Ng Y., McClelland J.F. (2008). Monitoring Ambient-Temperature Aging of a Carbon-Fiber/Epoxy Composite Prepreg with Photoacoustic Spectroscopy. Compos. Part A Appl. Sci. Manuf..

[20] Ahn K.J., Seferis J.C., Pelton T., Wilhelm M. (1992). Deformation Parameters Influencing Prepreg Tack. SAMPE Q. Soc. Aerosp. Mater. Process Eng. States.

[21] Shin Y., Qiao Y., Ni Y., Ramos J.L., Nickerson E.K., Merkel D.R., Simmons K.L. (2023). Interfacial Bond Characterization of Epoxy Adhesives to Aluminum Alloy and Carbon Fiber-Reinforced Polyamide by Vibrational Spectroscopy. Surf. Interfaces.

[22] Semoto T., Tsuji Y., Yoshizawa K. (2011). Molecular Understanding of the Adhesive Force between a Metal Oxide Surface and an Epoxy Resin. J. Phys. Chem. C.

[23] Voyutskii S.S., Vakula V.L. (1963). The Role of Diffusion Phenomena in Polymer-to-polymer Adhesion. J. Appl. Polym. Sci..

[24] Budelmann D., Schmidt C., Meiners D. (2021). Adhesion-Cohesion Balance of Prepreg Tack in Thermoset Automated Fiber Placement. Part 1: Adhesion and Surface Wetting. Compos. Part C Open Access.

[25] Wool R.P., O’Connor K.M. (1981). A Theory of Crack Healing in Polymers. J. Appl. Phys..

[26] De Gennes P.G. (1971). Reptation of a Polymer Chain in the Presence of Fixed Obstacles. J. Chem. Phys..

[27] Edwards S.F. (1967). The Statistical Mechanics of Polymerized Material. Proc. Phys. Soc..

[28] Janković B. (2018). Kinetic and Reactivity Distribution Behaviors during Curing Process of Carbon/Epoxy Composite with Thermoplastic Interface Coatings (T800/3900-2 Prepreg) under the Nonisothermal Conditions. Polym. Compos..

[29] Ma X.Q., Gu Y.Z., Li M., Li Y.X., Zhang D.M., Jia L.J., Zhang Z.G. (2013). Properties of Carbon Fiber Composite Laminates Fabricated by Coresin Film Infusion Process for Different Prepreg Materials. Polym. Compos..

